# Explainable machine learning identifies knee morphology thresholds for arthroscopic medial meniscus posterior root tear: a retrospective cohort study

**DOI:** 10.3389/fmed.2026.1819067

**Published:** 2026-05-14

**Authors:** Minyuan Zhang, Fengyuan Guo, Yanlin Li, Jiali Zheng, Yang Yu, Miao Chen, Runze Li, Yatong Liao, Qianzeng Chen, Chuan He

**Affiliations:** Department of Sports Medicine, The First Affiliated Hospital of Kunming Medical University, Kunming, China

**Keywords:** machine learning, medial meniscus posterior root tear, medial tibial plateau depth, medial tibial slope, MRI

## Abstract

**Background:**

Medial meniscus posterior root tear (MMPRT) is clinically important because disruption of posterior root function compromises hoop tension and load sharing, accelerating medial-compartment degeneration. Early or subtle posterior pathology may be underrecognized on MRI, highlighting the potential value of morphology-based risk awareness during MRI interpretation.

**Methods:**

We retrospectively analyzed 608 patients who underwent arthroscopic surgery for knee joint injuries, including 281 patients with arthroscopically confirmed MMPRT and 327 controls without MMPRT. Demographic, clinical, and MRI-based morphologic parameters were compared in the training set. Variables identified from training-set comparisons and clinical/biomechanical relevance were used to develop 10 machine learning models, including CatBoost, Decision Tree, GBM, LightGBM, LASSO, Naive Bayes, Neural Network, Random Forest, Support Vector Machine, and XGBoost. Models were evaluated with 10-fold cross-validation and an independent testing set. Explainability was assessed using SHapley Additive exPlanations (SHAP), including global importance and dependence plots.

**Results:**

Training-set multivariable analysis identified older age, greater medial tibial slope (MTS), and deeper medial tibial plateau depth (MTPD) as independent factors associated with MMPRT. GBM achieved the highest AUC in the independent testing set and was selected for SHAP-based interpretation. SHAP analysis ranked age as the dominant contributor, followed by MTS and MTPD. Dependence plots suggested non-linear, threshold-like patterns in model contribution. Age showed an apparent transition from negative to positive SHAP contributions around midlife, MTS showed a threshold-like increase between approximately 6° and 8°, whereas MTPD shifted toward positive SHAP contributions around approximately 2.2–2.5 mm.

**Conclusion:**

Age was the dominant model contributor, and MTS and MTPD were independently associated with MMPRT, exhibiting non-linear patterns in SHAP-based model interpretation. These findings may help raise suspicion for MMPRT in symptomatic patients undergoing MRI, particularly when age-related and tibial plateau morphologic risk patterns are present.

## 1 Introduction

Medial meniscus posterior root tear (MMPRT), involving the posterior root and its adjacent attachment complex, has gained recognition as a clinically important lesion because disruption of the meniscal root complex compromises hoop stress transmission, increases joint contact stress, and is linked to rapid medial compartment degeneration (1). Accurate early recognition remains challenging; although magnetic resonance imaging (MRI) is the primary non-invasive modality for evaluating meniscal pathology, subtle or early posterior root injuries may be underdetected due to complex tear morphology, limited visualization of the meniscotibial attachments, and the static nature of routine imaging relative to dynamic loading conditions (1). Consequently, there is a practical need to identify structural phenotypes associated with MMPRT in symptomatic patients, particularly when MRI does not yet show a definitive tear configuration, so as to raise diagnostic suspicion and support more careful image interpretation.

Accumulating evidence suggests that MMPRT is influenced not only by symptoms or isolated traumatic events but also by tibiofemoral geometry and the biomechanical environment it generates during weight-bearing flexion (2). In the sagittal plane, medial tibial slope (MTS) has been repeatedly associated with MMPRT, probably because a steeper posterior slope may increase shear-related loading on the posterior horn-root complex during weight-bearing flexion (2–4). Medial tibial plateau depth (MTPD), which reflects medial plateau concavity, may also be relevant because it can influence posterior compartment constraint and the local mechanical environment of the posterior root (5). However, most previous studies have relied primarily on conventional linear analyses or isolated cutoff-based comparisons, and less is known about whether MRI-based knee morphologic parameters show non-linear contribution patterns across their continuous ranges.

Traditional multivariable regression remains an important and interpretable baseline for identifying factors independently associated with binary outcomes, but it commonly relies on linear assumptions and may not fully capture non-linear relationships or changes in model contribution across the feature range. Explainable machine learning offers a complementary framework for exploring such patterns while improving model interpretability (6). SHapley Additive exPlanations (SHAP) provides both global and individualized attribution and enables visualization of non-linear dependence patterns, facilitating model interpretability and visualization of potential non-linear relationships (7). Therefore, this study aimed to identify clinical variables and MRI-based knee morphologic parameters associated with arthroscopically confirmed MMPRT, compare conventional multivariable logistic regression with multiple machine learning models under the same train–test framework, and use SHAP analysis to explore non-linear model-contribution patterns of key variables (7, 8).

## 2 Materials and methods

**FIGURE 1:**
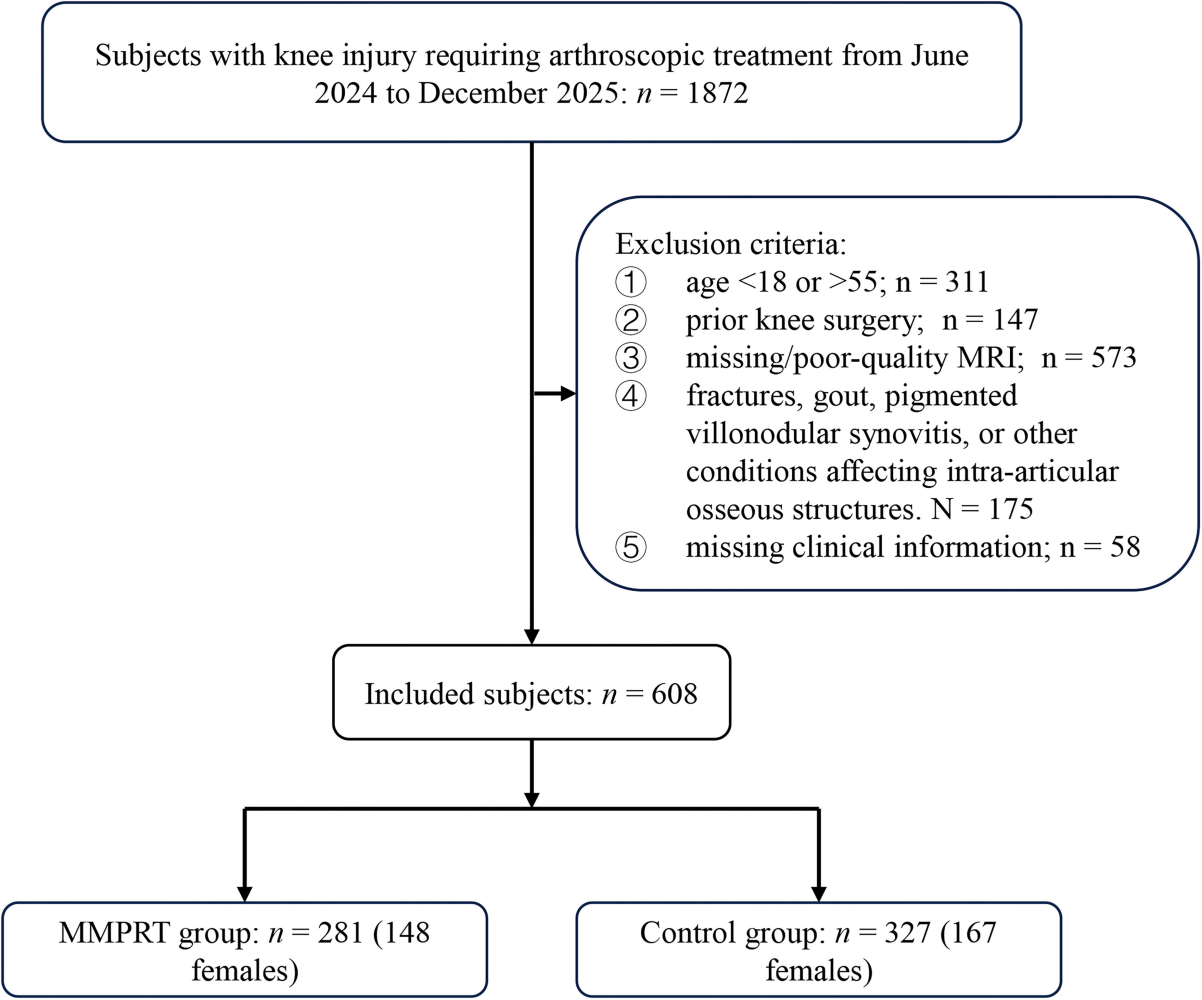
Flow diagram of the selection of eligible subjects.

This retrospective case-control study included 1872 patients who underwent arthroscopic surgery for knee joint injuries between June 2024 and December 2025. The inclusion criteria were as follows: patients with knee joint injuries confirmed by physical examination and MRI, requiring arthroscopic intervention. The exclusion criteria were: (1) age < 18 or > 55 years. Patients younger than 18 years were excluded to avoid the influence of incomplete skeletal development, whereas patients older than 55 years were excluded to reduce potential confounding from age-related osteoarthritic and degenerative structural changes (9, 10); (2) previous history of knee joint surgery; (3) missing preoperative MRI or MRI of insufficient quality, defined as severe motion artifacts, incomplete sagittal, coronal, or axial sequences, or inability to clearly identify the anatomical landmarks required for MRI-based morphologic measurements; (4) coexisting conditions such as knee fractures, gout, pigmented villonodular synovitis, or other diseases that could affect knee joint bone structure; (5) incomplete clinical information, defined as missing essential demographic information, BMI, arthroscopic findings, concomitant injury status, or key MRI-based morphologic measurements required for the present analysis. A total of 608 patients (293 males, 315 females) were included in the study. Patients with MMPRTs confirmed by intraoperative arthroscopic examination were assigned to the tear group (*n* = 281), while the remaining patients were assigned to the control group (*n* = 327) (Figure 1). In this study, we trained, evaluated, and compared 10 different types of machine learning models, including CatBoost, Decision Tree, GBM, LightGBM, LASSO, Naive Bayes, Neural Network, Random Forest, Support Vector Machine, and XGBoost, to evaluate factors associated with MMPRT and compare the performance of different machine learning models. This study was approved by our institutional review board and conducted in accordance with the ethical standards outlined in the 1964 Declaration of Helsinki.

### 2.1 Data collection

**FIGURE 2:**
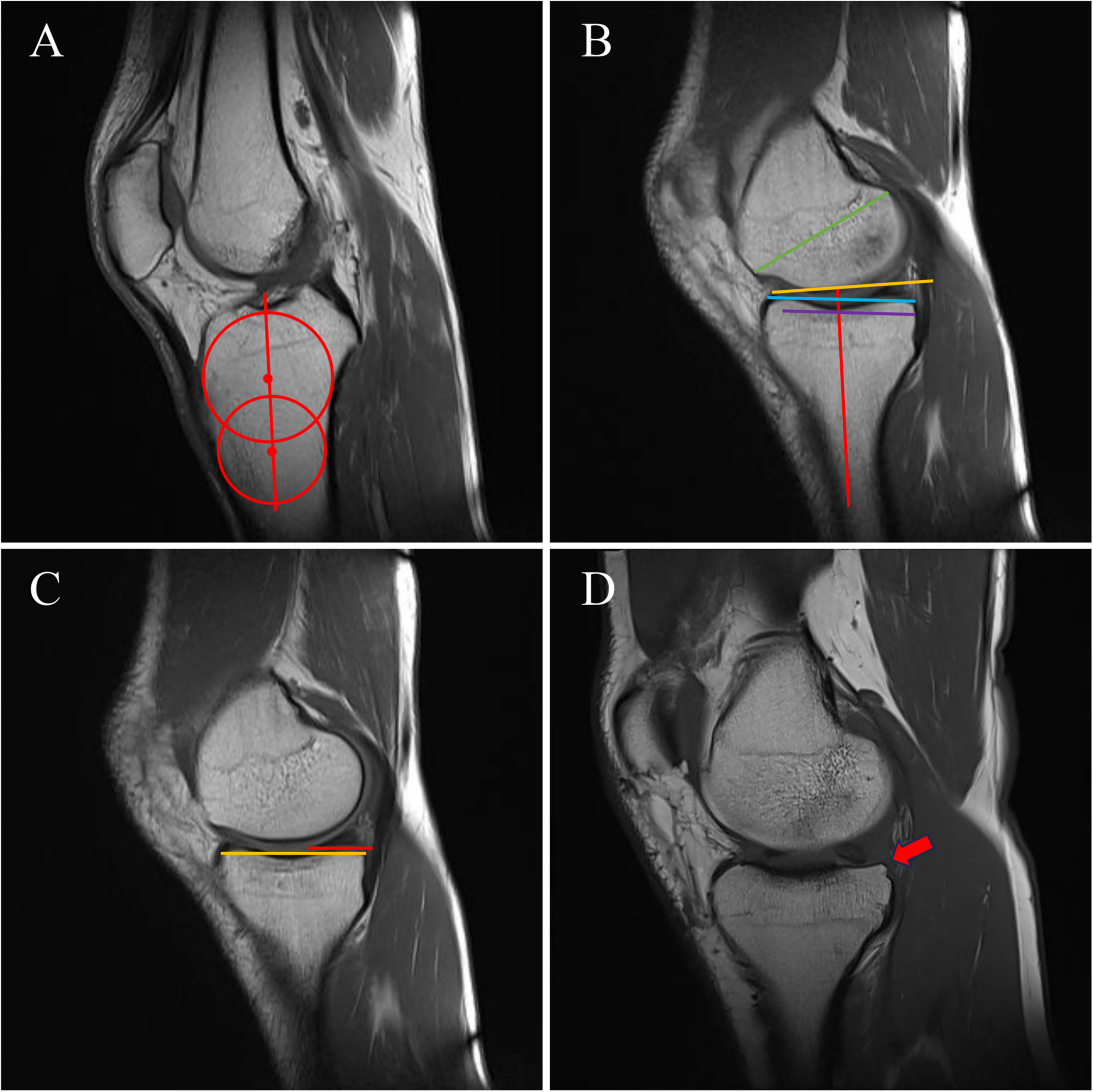
Measurement of osseous morphologic parameters on sagittal MRI. **(A)** Determination of the tibial anatomical axis (red line). **(B)** The MTS was calculated as *90*° *minus* the angle between the tibial anatomical axis and the blue line. The MTPD was defined as the perpendicular distance between the blue line and the purple line. The MMS was measured as the angle between the tibial anatomical axis and the yellow line. The MFCL was defined as the length of the green line, and the MTPL as the length of the blue line; the ratio MFCL/MTPL was then computed. **(C)** MMPHr was calculated as the ratio of the red-line length to the yellow-line length. **(D)** The red arrow indicates a posterior tibial osteophyte. MTS, medial tibial slope; MTPD, medial tibial plateau depth; MMS, medial meniscal slope; MFCL, medial femoral condyle length; MTPL, medial tibial plateau length; MMPHr, medial meniscus posterior root tibial coverage ratio.

Patient characteristics included age, sex, body mass index (BMI), laterality of the affected limb (left or right), and the presence of concomitant lateral meniscus, anterior cruciate ligament (ACL), or posterior cruciate ligament (PCL) injuries. MRI examinations were performed using a 3.0T machine (Magnetom Aera; Siemens) with an 8-channel knee coil, and slice thickness was set at 1.5 mm. A total of 10 MRI-based knee morphologic parameters were measured, including medial tibial slope (MTS), medial tibial plateau depth (MTPD), medial meniscal slope (MMS), the ratio of medial femoral condyle length (MFCL) to medial tibial plateau length (MTPL), medial meniscus posterior root tibial coverage ratio (MMPHr), posterior tibial osteophytes, femoral condylar notch shape, medial femoral condylar angle (MFCA), intercondylar notch width index (NWI) and lateral femoral condyle width index (LFCWI). The presence of damage to the medial and lateral menisci, as well as the cruciate ligaments, was confirmed by intraoperative arthroscopic examination to ensure the accuracy of the outcome variables and covariates. The measurement methods for the 10 knee joint bone structural features are as follows:

The MTS was measured on sagittal MRI images by first identifying the anatomical longitudinal axis of the tibia. Then, on the standard sagittal plane of the medial tibial plateau, a line was drawn along the horizontal surface of the medial plateau joint. MTS was calculated as 90° minus the angle between the tibial longitudinal axis and the platform joint surface line (11) (Figure 2).

The MTPD was measured on sagittal MRI images: On the standard sagittal plane of the medial tibial plateau, a baseline was formed by connecting the highest points of the anterior and posterior cortical margins of the tibial plateau. A tangent was drawn parallel to the baseline at the deepest point of the subchondral bone. The vertical distance between the baseline and the tangent was defined as MTPD (12) (Figure 2).

The MMS was measured on sagittal MRI images by first determining the anatomical axis of the proximal tibia. Then, on the standard sagittal plane at the center of the medial tibial plateau, a line was drawn connecting the anterior and posterior roots of the medial meniscus. The angle between this line and the anatomical axis of the proximal tibia was defined as MMS (13) (Figure 2).

The MFCL/MTPL was measured on MRI to characterize the bony alignment of the MFCL and the MTPL in the anteroposterior direction. The MFCL was measured on the standard sagittal plane as the maximum distance from the junction of the patella and the anterior part of the medial femoral condyle to the center point of the posterior joint surface of the femur. The MTPL was measured as the maximum anteroposterior diameter of the medial tibial plateau. The MFCL/MTPL was then calculated (14) (Figure 2).

The MMPHr was also measured on MRI. In the sagittal plane image, the distance from the insertion of the semimembranosus muscle to the anterior edge of the MMPH was measured to define the MMPH tibial coverage length. Simultaneously, the tibial width (TW) at the same plane was measured, and the coverage ratio was calculated as the coverage length divided by TW (15) (Figure 2).

Posterior tibial osteophytes were assessed on knee MRI by sequentially reviewing the posterior joint surface of the tibial plateau to identify any posteriorly protruding osteophytes. If an osteophyte was visible on the sagittal plane image and in contact with the posterior edge of the medial meniscus posterior root, it was classified as “present.” If no such contact was observed, it was classified as “absent” (15) (Figure 2).

**FIGURE 3:**
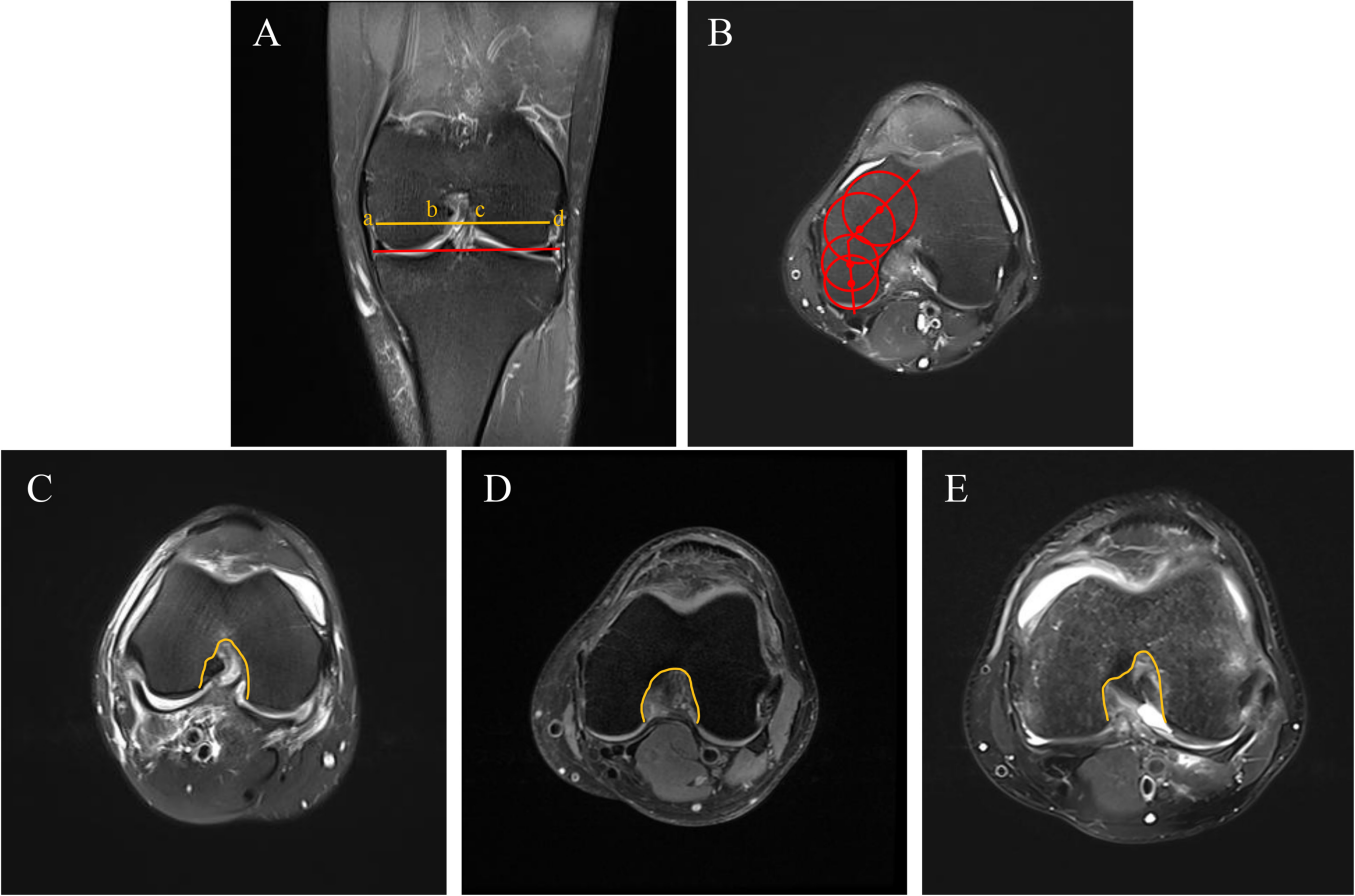
Measurement of osseous morphologic parameters on coronal and axial MRI. **(A)** The red line connects the distal ends of the medial and lateral femoral condyles. The yellow line is drawn at the level of the popliteal groove and parallel to the red line. Points *a* and *d* are defined at the medial and lateral distal condylar margins on the red line, and points *b* and *c* are defined at the medial and lateral borders of the intercondylar notch on the yellow line. The NWI was calculated as bc/ad, and the LFCWI as cd/ad. **(B)** The MFCA was defined as the angle formed by the two red lines. **(C–E)** Representative examples of intercondylar notch morphology: A-shaped **(C)**, U-shaped **(D)**, and W-shaped **(E)**. NWI, notch width index; LFCWI, lateral femoral condyle width index; MFCA, medial femoral condyle angle.

The MFCA was measured on axial MRI images at the level of the intercondylar region presenting a “Roman arch” shape. Two circles were placed at the anterior and posterior parts of the medial femoral condyle, both tangential to the cortical bone. The angle between the straight line connecting (or extending through) the centers of these two circles was defined as the MFCA (16). The shape of the femoral condylar notch (intercondylar notch morphology) was classified into three types—A, U, or W—based on established morphological criteria in the literature, evaluated at the same axial plane (17) (Figure 3).

The NWI and LFCWI were measured on coronal MRI images of the knee. A baseline was drawn by connecting the lowest points of the medial and lateral femoral condyles. A measurement line parallel to this baseline was drawn at the level of the popliteal groove. At this level, the total femoral condyle width (FCW), the notch width (NW), and the lateral femoral condyle width (LFCW) were measured. The intercondylar notch width index was calculated as NWI = NW/FCW, and the lateral femoral condyle width index was calculated as LFCWI = LFCW/FCW (18) (Figure 3).

We additionally reviewed the preoperative MRI reports of patients with arthroscopically confirmed MMPRT to determine how many cases had been explicitly identified as MMPRT before arthroscopy.

### 2.2 Statistical analysis

Data were analyzed using SPSS (version 27.0; IBM) and R (version 4.4.3; R Foundation). To avoid information leakage, the full dataset was first randomly divided into a training set and an independent testing set at a ratio of 7:3 before any univariable comparison, feature selection, regression analysis, or machine learning model development. The training set was used for statistical screening, multivariable regression analysis, model training, and cross-validation, whereas the independent testing set was held out and used only for final performance evaluation. There was no overlap between the two datasets.

Within the training set, differences between patients with and without medial meniscus posterior root tear were compared. Statistical significance was assessed using the Mann–Whitney U test, independent-samples *t*-test, or chi-square test, depending on the data distribution and variable type. Variables that showed significant differences between groups in the training set were further assessed using variance inflation factor (VIF) analysis to evaluate multicollinearity and prevent its potential impact on subsequent machine learning model training. Variables that exhibited significant group differences and had a VIF < 10 were then entered into multivariable logistic regression to identify factors independently associated with medial meniscus posterior root tear in the training set.

In addition, to assess the incremental value of machine learning relative to a conventional statistical approach, we constructed a standard multivariable logistic regression baseline model using the same variables selected for the final machine learning analyses. The baseline logistic regression model was fitted in the training set and then applied to the independent testing set to generate predicted probabilities. Its discriminative performance in the testing set was evaluated using the area under the receiver operating characteristic curve (AUC), accuracy, sensitivity, and specificity, so that its performance could be directly compared with that of the machine learning models under the same train–test split.

Based on the training-set between-group comparisons and the biological relevance of candidate predictors, variables were included in subsequent machine learning model development. These variables were selected before model training and were used consistently across all machine learning algorithms. The training set was used for model training and cross-validation to comprehensively assess the overall performance and stability of the models, preventing both overfitting and underfitting. We employed 10-fold cross-validation within the training set to build, train, and validate the models, providing an effective assessment of model reliability and stability. The independently separated testing set was then used only for final performance evaluation, assessing each model’s generalizability when faced with unseen data.

Sensitivity, specificity, accuracy, and F1 score for each model were calculated to provide a comprehensive evaluation of model performance and variability, aiding in the assessment of model stability. Receiver operating characteristic curves were used to evaluate the accuracy and consistency of the models’ predicted probabilities and to assess their discriminative performance at different thresholds.

### 2.3 Model explainability (SHAP)

SHAP (SHapley Additive exPlanations) was used to interpret the predictions of the final selected machine learning model. SHAP is based on Shapley values, a fair allocation method from game theory, and quantifies the contribution of each feature to the model prediction. After model development and performance comparison were completed, SHAP analysis was applied to the selected model to assess the relative importance and direction of contribution of each variable.

Global feature importance was evaluated using the mean absolute SHAP value. SHAP summary plots were used to visualize whether higher or lower feature values increased or decreased the predicted probability of medial meniscus posterior root tear. For key continuous variables, SHAP dependence plots were generated to explore potential non-linear or threshold-like associations between variable values and model output. This approach provided an intuitive interpretation of how key variables contributed to model-based prediction while preserving the revised leakage-free analytical workflow.

## 3 Results

### 3.1 Group comparisons in the training set

The differences in demographic and bone structural characteristics between patients with and without medial meniscus posterior root tear in the training set are shown in Table 1. Compared with the control group, the MMPRT group had a significantly higher age (40.36 ± 12.03 vs. 32.97 ± 11.30 years, *P* < 0.001), higher BMI (25.31 ± 3.75 vs. 24.35 ± 3.59 kg/m^2^, *P* = 0.008), greater MTS (9.32 ± 3.09° vs. 7.54 ± 3.14°, *P* < 0.001), deeper MTPD (2.62 ± 0.95 vs. 2.20 ± 0.95 mm, *P* < 0.001), smaller MMS (84.45 ± 3.79° vs. 85.93 ± 3.67°, *P* < 0.001), and a higher prevalence of posterior tibial osteophytes (24.2% vs. 8.6%, *P* < 0.001). Other clinical and MRI-based morphologic parameters did not differ significantly between groups, as shown in Table 1.

**TABLE 1:** Clinical and imaging characteristics in the training set.

Variable	MMPRT on arthroscopic examination		*P*-value
	No (*n* = 232)	Yes (*n* = 194)	
Age, year	32.97 ± 11.30	40.36 ± 12.03	**< 0.001**
Left knee, n	116 (50.0%)	104 (53.6%)	0.519
Lateral meniscus injury, n			0.954
No	131 (56.5%)	111 (57.2%)	
Yes	101 (43.5%)	83 (42.8%)	
ACL injury, n			0.342
No	142 (61.2%)	109 (56.2%)	
Yes	90 (38.8%)	85 (43.8%)	
PCL injury, n			0.207
No	217 (93.5%)	174 (89.7%)	
Yes	15 (6.5%)	20 (10.3%)	
Sex, n			0.729
Male	111 (47.8%)	97 (50.0%)	**0.008**
Female	121 (52.2%)	97 (50.0%)	
BMI, kg/m^2^	24.35 ± 3.59	25.31 ± 3.75	
MTS,°	7.54 ± 3.14	9.32 ± 3.09	**< 0.001**
MTPD, mm	2.20 ± 0.95	2.62 ± 0.95	**< 0.001**
MMS,°	85.93 ± 3.67	84.45 ± 3.79	**< 0.001**
MFCL/MTPL	1.12 ± 0.09	1.12 ± 0.08	0.935
MMPHr	0.39 ± 0.05	0.39 ± 0.06	0.887
Posterior Tibial Osteophytes, n			< 0.001
Present	20 (8.6%)	47 (24.2%)	
Absent	212 (91.4%)	147 (75.8%)	
Notch shape, n			0.706
A	169 (72.8%)	136 (70.1%)	0.722
U	59 (25.4%)	53 (27.3%)	
W	4 (1.7%)	5 (2.6%)	
MFCA,°	131.79 ± 5.82	132.01 ± 6.35	
NWI	0.26 ± 0.04	0.26 ± 0.04	0.760
LFCWI	0.39 ± 0.03	0.39 ± 0.03	0.486

Data are presented as *n* (%) or mean ± standard deviation. Values in bold indicate statistical significance (*P* < 0.05). MMPRT, medial meniscus posterior root tear; ACL, anterior cruciate ligament; PCL, posterior cruciate ligament; BMI, body mass index; MTS, medial tibial slope; MTPD, medial tibial plateau depth; MMS, medial meniscal slope; MFCL, medial femoral condyle length; MTPL, medial tibial plateau length; MMPHr, medial meniscus posterior root tibial coverage ratio; NWI, notch width index; LFCWI, lateral femoral condyle width index; MFCA, medial femoral condylar angle.

Boxplots illustrate the significant differences in continuous variables between the two groups, while stacked bar charts show the significant difference in posterior tibial osteophytes between the groups (Supplementary Figure 1).

Among the 281 patients with arthroscopically confirmed MMPRT, 223 patients (79.4%) had MMPRT explicitly reported on preoperative MRI, whereas 58 patients (20.6%) did not have MMPRT explicitly identified before arthroscopy.

### 3.2 Results of logistic regression analysis

Variables that showed statistically significant differences between the MMPRT and control groups in the training set were further entered into multivariable binary logistic regression analysis. The results showed that older age (*P* < 0.001), greater MTS (*P* < 0.001), and deeper MTPD (*P* = 0.008) were independently associated with MMPRT in the training set. In contrast, BMI (*P* = 0.052), MMS (*P* = 0.263), and posterior tibial osteophytes (*P* = 0.131) were not independently associated with MMPRT after multivariable adjustment (Table 2).

**TABLE 2:** Training-set multivariable binary logistic regression analysis of factors associated with MMPRT.

Variable	*P*-value	Odds ratio	95% CI for odds ratio	
			Lower	Upper
Age	**< 0.001**	1.047	1.027	1.066
BMI	0.052	1.060	1.000	1.125
MTS	**< 0.001**	1.200	1.083	1.329
MTPD	**0.008**	1.367	1.084	1.723
MMS	0.263	1.050	0.964	1.143
Posterior Tibial Osteophytes	0.131	1.631	0.864	3.076

Results are derived from multivariable binary logistic regression models performed in the training set and are reported as *P*-values, odds ratios (ORs), and 95% confidence intervals (CIs). For posterior tibial osteophytes, the odds ratio represents the comparison of patients with posterior tibial osteophytes versus those without posterior tibial osteophytes. Values in bold indicate statistical significance (*P* < 0.05). MMPRT, medial meniscus posterior root tear; BMI, body mass index; MTS, medial tibial slope; MTPD, medial tibial plateau depth; MMS, medial meniscal slope; OR, odds ratio; CI, confidence interval.

### 3.3 Model development and comparison

Based on the results of the training-set between-group comparisons and the biological relevance of candidate variables, six variables—age, BMI, MTS, MTPD, MMS, and posterior tibial osteophytes—were included in subsequent machine learning model development. Although not all variables remained statistically significant in the multivariable logistic regression analysis, they were retained because they represent clinically and biomechanically relevant demographic, anthropometric, and knee morphologic factors.

**FIGURE 4:**
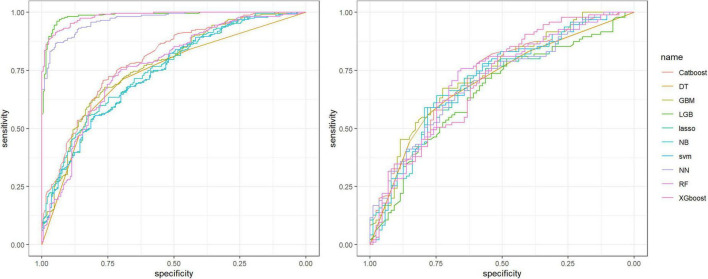
Discrimination performance of 10 models for classifying MMPRT status in the training and testing sets. Receiver operating characteristic (ROC) curves are shown for the training set (left panel) and the testing set (right panel). ROC, receiver operating characteristic; AUC, area under the ROC curve; MMPRT, medial meniscus posterior root tear; DT, decision tree; GBM, gradient boosting machine; LightGBM, Light Gradient Boosting Machine (LightGBM); LASSO, least absolute shrinkage and selection operator; NB, naive Bayes; NN, neural network; RF, random forest; XGBoost, extreme gradient boosting; SVM, support vector machine.

Ten machine learning algorithms were developed using the training set, including CatBoost, Decision Tree, GBM, LightGBM, LASSO, Naive Bayes, Neural Network, Random Forest, Support Vector Machine, and XGBoost. The AUC values and diagnostic performance metrics for each model in the training and testing sets are shown in Tables 3, 4, and the ROC curves are presented in Figure 4.

**TABLE 3:** AUC and diagnostic performance of different machine learning models in the training set.

Model	AUC	Accuracy	Sensitivity	Specificity	F1-score
CatBoost	0.798	0.728	0.747	0.711	0.714
DT	0.737	0.660	0.443	0.841	0.543
GBM	0.771	0.714	0.758	0.677	0.707
LGB	0.986	0.951	0.938	0.961	0.945
LASSO	0.740	0.676	0.675	0.677	0.655
NB	0.756	0.681	0.778	0.599	0.689
NN	0.740	0.676	0.670	0.681	0.653
RF	0.968	0.901	0.943	0.866	0.897
SVM	0.765	0.728	0.696	0.754	0.699
XGBoost	0.984	0.927	0.943	0.914	0.922

Model performance was evaluated in the training set. Sensitivity represents the true positive rate, and specificity represents the true negative rate. All values are reported to three decimal places. AUC, area under the receiver operating characteristic curve; DT, decision tree; GBM, gradient boosting machine; LGB, Light Gradient Boosting Machine; LASSO, least absolute shrinkage and selection operator; NB, Naive Bayes; NN, neural network; RF, random forest; SVM, support vector machine; XGBoost, Extreme Gradient Boosting; F1, F1 score.

**TABLE 4:** AUC and diagnostic performance of different machine learning models in the testing set.

Model	AUC	Accuracy	Sensitivity	Specificity	F1-score
CatBoost	0.723	0.665	0.701	0.632	0.667
DT	0.702	0.621	0.402	0.821	0.504
GBM	0.732	0.670	0.655	0.684	0.655
LGB	0.656	0.626	0.575	0.674	0.595
LASSO	0.705	0.648	0.621	0.674	0.628
NB	0.716	0.670	0.747	0.600	0.684
NN	0.706	0.648	0.621	0.674	0.628
RF	0.718	0.681	0.667	0.695	0.667
SVM	0.706	0.659	0.609	0.705	0.631
XGBoost	0.707	0.637	0.609	0.663	0.616

Model performance was evaluated in the independent testing set, which was not used for variable screening or model training. Sensitivity represents the true positive rate, and specificity represents the true negative rate. All values are reported to three decimal places. AUC, area under the receiver operating characteristic curve; DT, decision tree; GBM, gradient boosting machine; LGB, Light Gradient Boosting Machine; LASSO, least absolute shrinkage and selection operator; NB, Naive Bayes; NN, neural network; RF, random forest; SVM, support vector machine; XGBoost, Extreme Gradient Boosting; F1, F1 score.

In the training set, LightGBM, XGBoost, and Random Forest showed the highest apparent discrimination, with AUC values of 0.986, 0.984, and 0.968, respectively. However, their performance decreased substantially in the independent testing set, with corresponding AUC values of 0.656, 0.707, and 0.718, suggesting potential overfitting. In contrast, GBM demonstrated the highest AUC in the independent testing set (AUC = 0.732) while maintaining relatively consistent discrimination between the training set (AUC = 0.771) and testing set.

In the independent testing set, AUC values ranged from 0.656 to 0.732 across the evaluated models. Considering both testing-set AUC and the difference between training-set and testing-set performance, GBM was selected as the primary model for subsequent interpretability analysis.

### 3.4 Comparison with conventional logistic regression

To compare the machine learning framework with a conventional statistical baseline, we additionally fitted a standard multivariable logistic regression model in the training set using the same six variables included in the machine learning analyses: age, BMI, MTS, MTPD, MMS, and posterior tibial osteophytes. The fitted logistic regression model was then applied to the independent testing set for performance evaluation. In the testing set, the logistic regression model achieved an AUC of 0.706, with an accuracy of 0.654, sensitivity of 0.563 and specificity of 0.737 (Supplementary Table 1).

Among the machine learning models, GBM achieved an AUC of 0.771 in the training set and 0.732 in the independent testing set. In the testing set, GBM showed an accuracy of 0.670, sensitivity of 0.655, specificity of 0.684, and F1 score of 0.655. Compared with the logistic regression model, GBM showed higher AUC, accuracy, sensitivity, and F1 score, whereas the logistic regression model showed higher specificity.

### 3.5 Model explainability (SHAP)

**FIGURE 5:**
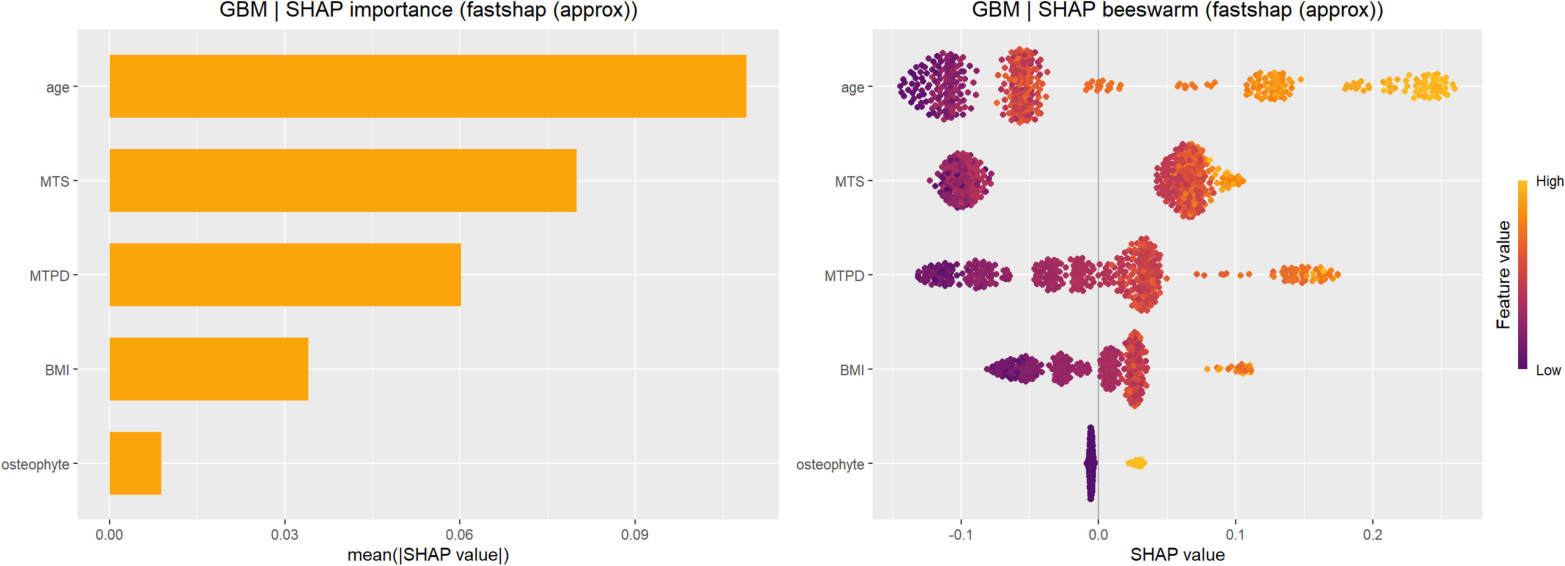
SHAP-based interpretation of the GBM model for classifying MMPRT status. Global feature importance ranked by the mean absolute SHAP value [mean(|SHAP|)], indicating each variable’s average contribution to the GBM model output across all individuals (left). SHAP summary (beeswarm) plot showing the distribution of SHAP values for each variable; each dot represents one patient (right). Positive SHAP values indicate an increased model-predicted probability of MMPRT, whereas negative SHAP values indicate a decreased model-predicted probability. Dot color encodes the original feature value from low to high. The model identified age as the most influential variable, followed by MTS, MTPD, BMI, and posterior tibial osteophyte. MMS showed negligible contribution and is not displayed in the global importance ranking. SHAP, Shapley additive explanations; GBM, gradient boosting machine; MMPRT, medial meniscus posterior root tear; MTS, medial tibial slope; MTPD, medial tibial plateau depth; BMI, body mass index.

In the final model comparison, GBM was selected as the primary model for interpretability analysis, and SHAP was used to decompose the model outputs. Based on the global SHAP importance ranking, age contributed the most to the model prediction, followed by MTS and MTPD. BMI showed a smaller contribution, whereas posterior tibial osteophytes and MMS had relatively limited contributions to the model output. In the SHAP beeswarm plot, higher values of age, MTS, and MTPD generally tended to produce positive SHAP values, suggesting that these features were associated with an increased model-predicted probability of medial meniscus posterior root tear. Conversely, lower values of these variables were predominantly associated with negative SHAP values, indicating a lower model-predicted probability of the outcome (Figure 5).

**FIGURE 6:**
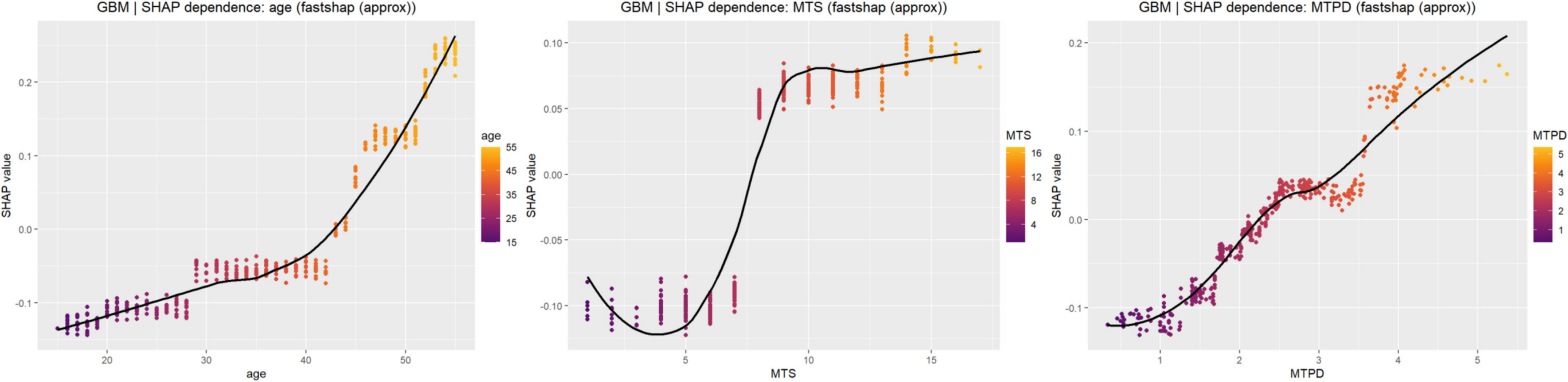
SHAP dependence plots for key variables in the GBM model. SHAP dependence plots illustrate the marginal contribution patterns of age (left), MTS (middle), and MTPD (right) to the GBM-estimated probability of MMPRT. The solid black curve represents a smoothed trend capturing potential non-linear or threshold-like patterns. Point color encodes the corresponding feature value from low to high to aid interpretation of the distribution across the variable range. SHAP, Shapley additive explanations; GBM, gradient boosting machine; MMPRT, medial meniscus posterior root tear; MTS, medial tibial slope; MTPD, medial tibial plateau depth.

Through SHAP dependence plot analysis of the three most important continuous variables in the GBM model, we observed non-linear SHAP patterns for age, MTS, and MTPD, with apparent transition ranges in model contribution. Age showed a transition from negative to positive SHAP contributions around the mid-40 s, followed by increasingly positive contributions at older ages. MTS demonstrated a threshold-like increase in SHAP contribution between approximately 6° and 8°, after which SHAP values generally remained positive. MTPD showed a gradual non-linear increase, with SHAP values shifting toward positive contributions around approximately 2.2–2.5 mm and increasing further at higher values (Figure 6).

Overall, the SHAP interpretation results indicated that age, MTS, and MTPD were the primary model contributors associated with higher GBM-estimated probability of MMPRT. Their SHAP contributions showed non-linear patterns, with more positive contributions beyond specific value ranges.

## 4 Discussion

### 4.1 Principal findings and overall interpretation

This study evaluated clinical and MRI-based osseous morphologic factors associated with arthroscopically confirmed medial meniscus posterior root tear (MMPRT) using a leakage-free train–test analytical framework. After the full dataset was first divided into training and independent testing sets, all between-group comparisons, multivariable regression analyses, and machine learning model development were performed within the training set. In the training-set multivariable analysis, older age, greater medial tibial slope (MTS), and deeper medial tibial plateau depth (MTPD) were independently associated with MMPRT. Among the evaluated machine learning algorithms, GBM achieved the highest AUC in the independent testing set and was selected for SHAP-based interpretation.

These findings are consistent with the recognition that MMPRT is not merely an isolated meniscal lesion, but a pathology related to patient background, tibiofemoral morphology, and medial-compartment loading. Meniscus root tears alter knee biomechanics, increase compartmental contact pressure, and may contribute to progressive joint degeneration if not recognized appropriately (19). SHAP analysis further identified age, MTS, and MTPD as the leading contributors to the GBM model. The strong contribution of age is clinically expected because older age reflects degenerative vulnerability and reduced tolerance of meniscal tissue to repetitive loading. Therefore, the following discussion focuses mainly on MTS, MTPD, and the added interpretability provided by machine learning.

### 4.2 MTS and shear-related posterior root loading

MTS was the most important osseous morphologic variable in this study. Patients with MMPRT had greater MTS in the training set, and MTS remained independently associated with MMPRT after multivariable adjustment. In the GBM-SHAP analysis, MTS ranked second only to age. This finding is consistent with recent systematic reviews and clinical studies showing that increased posterior tibial slope, especially increased medial tibial slope, is associated with meniscal root tears or MMPRT (2, 3, 8).

Biomechanically, a steeper medial tibial slope may increase shear-related loading across the tibiofemoral joint during weight-bearing flexion. Because the medial meniscus posterior root maintains hoop tension and load transmission, increased slope-related shear may place greater stabilizing demands on the posterior horn-root complex. Recent biomechanical evidence supports this mechanism, showing that increased posterior tibial slope increases force on the posterior medial meniscus root and affects compressive and shear-related loading at the root complex (4).

The SHAP dependence plot suggested that the contribution of MTS was not strictly linear. Lower MTS values were generally associated with negative SHAP values, whereas SHAP contributions increased rapidly between approximately 6° and 8° and remained mostly positive thereafter. This range is broadly consistent with a recent anatomical risk-factor study reporting an MTS cutoff of 7.4° for MMPRT discrimination (8). However, the 6°–8° range in this study should be interpreted as a model-derived transition in SHAP contribution, not as a validated clinical cutoff or direct biomechanical threshold.

### 4.3 MTPD and medial tibial plateau constraint

MTPD was another key morphologic factor associated with MMPRT. In the training set, MTPD was greater in the MMPRT group and remained independently associated with MMPRT in multivariable logistic regression. In the GBM-SHAP analysis, MTPD ranked third, suggesting that medial plateau concavity provided additional model-relevant information beyond slope alone.

MTPD reflects the sagittal-plane concavity of the medial tibial plateau and may influence posterior compartment constraint during knee flexion and loading. A deeper medial plateau may alter the posterior medial contact environment and affect deformation of the medial meniscus posterior horn-root complex under compressive and shear forces. Recent clinical work in surgically treated isolated meniscal tears reported larger medial tibial depth in patients with meniscal tears, supporting the possibility that a more concave medial plateau may be associated with some meniscal injury phenotypes (5). However, plateau-depth effects may vary across lesion type, degenerative background, alignment, and loading condition. Finite-element work has also shown that alignment and loading conditions can substantially influence medial compartment contact biomechanics after MMPRT repair, supporting the concept that osseous geometry and mechanical environment should be interpreted together (20).

The SHAP dependence plot showed that MTPD shifted toward positive contribution around approximately 2.2–2.5 mm and increased further at higher values. This suggests a non-linear, value-dependent model contribution. Nevertheless, this pattern should be regarded as hypothesis-generating rather than evidence of a definitive structural threshold.

### 4.4 Secondary role of BMI, MMS, and posterior tibial osteophytes

BMI, MMS, and posterior tibial osteophytes showed significant between-group differences in the training set but were not independently associated with MMPRT after multivariable adjustment. They were retained in the machine learning models because feature selection should not rely solely on a single regression *P*-value, especially when variables have plausible clinical or biomechanical relevance.

BMI reflects body habitus and loading background. Although BMI was not an independent factor in the present analysis, weight-related factors may influence MMPRT healing and medial meniscal extrusion after repair (21), and occupational kneeling and squatting have been associated with increased MMPRT development (22). MMS may reflect meniscal orientation, but its limited SHAP contribution suggests overlap with stronger tibial morphologic variables such as MTS and MTPD. Posterior tibial osteophytes were more common in the MMPRT group but were not independently associated after adjustment, suggesting a degenerative or impingement-related background rather than an independent driver. This is consistent with studies linking posteromedial impinging structures, including posterior tibial osteophytes, with medial meniscus posterior horn tears and associating meniscal extrusion with osteophyte formation in early knee osteoarthritis (15, 23).

### 4.5 Comparative performance and interpretability of machine learning and logistic regression

The comparison with conventional logistic regression provides important context. Using the same six variables, logistic regression achieved an AUC of 0.706 in the independent testing set, with an accuracy of 0.654, sensitivity of 0.563, specificity of 0.737, and F1 score of 0.609. GBM achieved an AUC of 0.732, accuracy of 0.670, sensitivity of 0.655, specificity of 0.684, and F1 score of 0.655. Thus, GBM showed higher AUC, accuracy, sensitivity, and F1 score, whereas logistic regression showed higher specificity.

The improvement in discrimination was modest. Therefore, machine learning should not be interpreted as substantially outperforming conventional regression in this dataset. This is consistent with current prediction-model reporting guidance, which emphasizes transparent evaluation of both regression and machine learning models rather than assuming algorithmic superiority (24). The main value of GBM was its ability to capture non-linear model-contribution patterns. When combined with SHAP, the model provided global feature importance and variable-specific dependence plots, allowing visualization of how age, MTS, and MTPD contributed across their observed ranges. SHAP has been recommended as a practical method for explaining supervised machine learning models and visualizing feature contribution patterns (7). This interpretability advantage is important because recent AI studies in meniscal MRI highlight that favorable internal performance does not automatically translate into clinical utility without validation and transparent interpretation (25–27).

### 4.6 Clinical implications and interpretation boundaries

In this cohort, MMPRT was explicitly reported on routine preoperative MRI in 223 of 281 arthroscopically confirmed cases (79.4%), indicating that a subset of tears may not be clearly identified before arthroscopy. These findings may be clinically relevant for symptomatic patients undergoing knee MRI, particularly when posterior root pathology is subtle or equivocal. Older age, greater MTS, and deeper MTPD may serve as imaging-based risk-awareness signals that prompt closer inspection of the medial meniscus posterior root region, meniscal extrusion, ghost signs, truncation signs, or subtle posterior horn abnormalities. However, the GBM-SHAP model should not be used as a stand-alone diagnostic or prognostic tool. The model was developed in a surgically treated retrospective cohort, and the SHAP-derived transition ranges for MTS and MTPD represent model-contribution patterns rather than validated cutoffs. Future studies should test whether these patterns are reproducible in external cohorts and prospective knee-pain populations.

### 4.7 Limitations and future directions

Several limitations should be acknowledged. First, this retrospective study of surgically treated patients limits causal inference and may introduce selection bias. The findings may not represent the full spectrum of symptomatic knee patients or individuals undergoing MRI in general practice. Therefore, these findings should not be directly generalized to the general population, primary MRI screening settings, or older patients with degenerative knee disease, particularly those with typical age-related degenerative MMPRT. Second, although an independent testing set was used to reduce information leakage, it was internally derived from the same cohort rather than from an external institution. External validation is therefore required, particularly because AI and prediction-model literature emphasizes that internal performance alone is insufficient to establish generalizability (24, 25). Third, model performance was moderate, and GBM provided only modest improvement over logistic regression. In addition, the absolute SHAP magnitudes were relatively small, indicating that the contribution of each individual variable to the model output was limited. Fourth, SHAP explains model behavior rather than causality; therefore, the 6°–8° MTS range and 2.2–2.5 mm MTPD range should not be considered validated clinical thresholds. Finally, residual confounding remains possible because lower-limb alignment, occupational loading, meniscal extrusion, cartilage status, sports exposure, and activity level were not fully incorporated. Future multicenter studies should combine standardized MRI measurements, external validation, and complementary non-linear methods such as restricted cubic splines or segmented regression.

## 5 Conclusion

In conclusion, older age, greater MTS, and deeper MTPD were independently associated with arthroscopically confirmed MMPRT. GBM showed the highest testing-set AUC among the evaluated machine learning models and provided SHAP-based visualization of non-linear contribution patterns, particularly for MTS and MTPD. These findings support the complementary value of combining conventional regression, machine learning, and explainability analysis to characterize MMPRT-associated knee morphology, while emphasizing the need for external validation before clinical application.

## Data Availability

The raw data supporting the conclusions of this article will be made available by the authors, without undue reservation.

## Supplementary material

Supplementary Figure 1 Visualization of variables showing statistically significant between-group differences based on the independent-samples *t*-test and the chi-square test. Box-and-whisker plots were used to display between-group distributions of continuous variables showing statistically significant differences in the training set (age, BMI, MTS, MTPD, and MMS), whereas a stacked bar chart was used to compare posterior tibial osteophytes between groups. BMI, body mass index; MTS, medial tibial slope; MTPD, medial tibial plateau depth; MMS, medial meniscal slope.

## References

[1] Guimarães J Chemin R Araujo F Link T Silva F Bitar A et al. Meniscal root tears: an update focused on preoperative and postoperative MRI findings. *AJR Am J Roentgenol.* (2022) 219:269–78. 10.2214/AJR.22.27338 35293231

[2] Oeding J Dean M Hevesi M Chahla J Krych A. Steeper slope of the medial tibial plateau, greater varus alignment, and narrower intercondylar distance and notch width increase risk for medial meniscus posterior root tears: a systematic review. *Arthroscopy.* (2025) 41:3172–83.e3. 10.1016/j.arthro.2024.10.031 39505159

[3] Dzidzishvili L Allende F Allahabadi S Mowers C Cotter E Chahla J. Increased posterior tibial slope is associated with increased risk of meniscal root tears: a systematic review. *Am J Sports Med.* (2024) 52:3427–35. 10.1177/03635465231225981 38362610

[4] Melugin H Brown J Hollenbeck J Fossum B Whalen R Ganokroj P et al. Increased posterior tibial slope increases force on the posterior medial meniscus root. *Am J Sports Med.* (2023) 51:3197–203. 10.1177/03635465231195841 37715505

[5] Barnett S Portila G Sanborn R Perone G Emami A Kiapour A. Comparison of size of posterior tibial slope and medial tibial depth in patients with an isolated meniscal tear requiring surgery and matched uninjured controls. *Am J Sports Med.* (2023) 51:3706–13. 10.1177/03635465231204362 37924211

[6] Allgaier J Mulansky L Draelos R Pryss R. How does the model make predictions? A systematic literature review on the explainability power of machine learning in healthcare. *Artif Intell Med.* (2023) 143:102616. 10.1016/j.artmed.2023.102616 37673561

[7] Ponce-Bobadilla A Schmitt V Maier C Mensing S Stodtmann S. Practical guide to SHAP analysis: explaining supervised machine learning model predictions in drug development. *Clin Transl Sci.* (2024) 17:e70056. 10.1111/cts.70056 39463176 PMC11513550

[8] Si N Hongbo L Jingping G Jiayu H Min L. Association between anatomical risk factors and medial meniscus posterior root tears: a retrospective study. *BMC Musculoskelet Disord.* (2025) 26:455. 10.1186/s12891-025-08676-y 40346501 PMC12063313

[9] Han X Zhang C Guan B Zhou H Kong X Feng S. Burden of osteoarthritis in older adults (aged ≥55 years) in the United States and China: a comparative analysis of temporal trends, risk factor contributions, and projected burden to 2030 using global burden of disease study 2021 data. *Front Med.* (2025) 12:1636976. 10.3389/fmed.2025.1636976 41035869 PMC12481515

[10] Martel-Pelletier J Pelletier J. Next-level prediction of structural progression in knee osteoarthritis: a perspective. *Int J Mol Sci.* (2025) 26:4748. 10.3390/ijms26104748 40429891 PMC12112129

[11] Hudek R Schmutz S Regenfelder F Fuchs B Koch P. Novel measurement technique of the tibial slope on conventional MRI. *Clin Orthop Relat Res.* (2009) 467:2066–72. 10.1007/s11999-009-0711-3 19190973 PMC2706341

[12] Hashemi J Chandrashekar N Gill B Beynnon B Slauterbeck J Schutt R et al. The geometry of the tibial plateau and its influence on the biomechanics of the tibiofemoral joint. *J Bone Joint Surg Am.* (2008) 90:2724–34. 10.2106/JBJS.G.01358 19047719 PMC2663332

[13] Li W Liang J Zeng F Lin B Liu C Huang S et al. Anatomic characteristics of the knee influence the risk of suffering an isolated meniscal injury and the risk factors differ between women and men. *Knee Surg Sports Traumatol Arthrosc.* (2021) 29:3751–62. 10.1007/s00167-020-06396-5 33388828

[14] Musahl V Ayeni O Citak M Irrgang J Pearle A Wickiewicz T. The influence of bony morphology on the magnitude of the pivot shift. *Knee Surg Sports Traumatol Arthrosc.* (2010) 18:1232–8. 10.1007/s00167-010-1129-x 20376621

[15] Lim S Chung J Park J Yun H Noh S Park D. Medial meniscus posterior horn horizontal tears are associated with knee posteromedial impinging structures inducing shearing forces in patients with meniscus degeneration. *Cartilage.* (2025): Online ahead of print 10.1177/19476035251347728 40605817 PMC12226532

[16] Altinayak H Karatekin Y. Increased medial femoral condyle angle and narrow intercondylar notch are associated with medial meniscus posterior root tear. *Arthroscopy.* (2023) 39:2154–63. 10.1016/j.arthro.2023.02.020 36868529

[17] van Eck C Martins C Vyas S Celentano U van Dijk C Fu F. Femoral intercondylar notch shape and dimensions in ACL-injured patients. *Knee Surg Sports Traumatol Arthrosc.* (2010) 18:1257–62. 10.1007/s00167-010-1135-z 20390246

[18] Souryal T Moore H Evans J. Bilaterality in anterior cruciate ligament injuries: associated intercondylar notch stenosis. *Am J Sports Med.* (1988) 16:449–54. 10.1177/036354658801600504 3189676

[19] Garcia J Ayala S Allende F Mameri E Haynes M Familiari F et al. Diagnosis and treatment strategies of meniscus root tears: a scoping review. *Orthop J Sports Med.* (2024) 12:23259671241283962. 10.1177/23259671241283962 39493310 PMC11531027

[20] Berk A Cregar W Wang S Habet N Ifarraguerri A Trofa D et al. The effect of lower limb alignment on tibiofemoral joint contact biomechanics after medial meniscus posterior root repair: a finite-element analysis. *J Am Acad Orthop Surg.* (2024) 32:e558–67. 10.5435/JAAOS-D-23-00702 38669669

[21] Hiranaka T Furumatsu T Yokoyama Y Higashihara N Tamura M Kawada K et al. Weight loss enhances meniscal healing following transtibial pullout repair for medial meniscus posterior root tears. *Knee Surg Sports Traumatol Arthrosc.* (2024) 32:143–50. 10.1002/ksa.12037 38226719

[22] Kawada K Yokoyama Y Tamura M Okazaki Y Ozaki T Furumatsu T. Occupational motions such as kneeling and squatting are associated with the increased development of medial meniscus posterior root tears, regardless of the medial posterior tibial slope angle. *J Exp Orthop.* (2025) 12:e70276. 10.1002/jeo2.70276 40390861 PMC12086786

[23] Fujita K Takata Y Goshima K Kanayama T Takemoto N Nishimura M et al. Relationship between medial meniscus extrusion and osteophyte formation in early-stage knee osteoarthritis: an ultrasound-based multicenter cross-sectional study. *J Med Ultrason.* (2025): Online ahead of print 10.1007/s10396-025-01599-0 41364182

[24] Collins G Moons K Dhiman P Riley R Beam A Van Calster B et al. TRIPOD+AI statement: updated guidance for reporting clinical prediction models that use regression or machine learning methods. *BMJ.* (2024) 385:e078378. 10.1136/bmj-2023-078378 38626948 PMC11019967

[25] Zhao Y Coppola A Karamchandani U Amiras D Gupte C. Artificial intelligence applied to magnetic resonance imaging reliably detects the presence, but not the location, of meniscus tears: a systematic review and meta-analysis. *Eur Radiol.* (2024) 34:5954–64. 10.1007/s00330-024-10625-7 38386028 PMC11364796

[26] Mohammadi S Jahanshahi A Shahrabi Farahani M Salehi M Frounchi N Guermazi A. Diagnosis of knee meniscal injuries using artificial intelligence: a systematic review and meta-analysis of diagnostic performance. *PLoS One.* (2025) 20:e0326339. 10.1371/journal.pone.0326339 40554500 PMC12186967

[27] Oeding J Krych A Pearle A Kelly B Kunze K. Medical imaging applications developed using artificial intelligence demonstrate high internal validity yet are limited in scope and lack external validation. *Arthroscopy.* (2025) 41:455–72. 10.1016/j.arthro.2024.01.043 38325497

