# Supplementary material for: Explainable machine learning identifies knee morphology thresholds for arthroscopic medial meniscus posterior root tear: a retrospective cohort study
Source: Front Med (Lausanne). 2026 May 14;13:1819067. doi: 10.3389/fmed.2026.1819067 (PMC13216056; doi:10.3389/fmed.2026.1819067)
Supplement: Supplementary file 1 [file Table_1.docx]

**Supplementary Table 1.** Standard multivariable logistic regression baseline model and its performance in the training and testing sets

**Panel A.** Standard multivariable logistic regression baseline model fitted in the training set

| **Variable** | **Coefficient β** | **Odds ratio OR** | **95% CI for OR** | **P value** |
| --- | --- | --- | --- | --- |
| Intercept | -9.798 | — | — | 0.019 |
| Age | 0.045 | 1.047 | 1.027–1.066 | <0.001 |
| BMI | 0.059 | 1.060 | 1.000–1.125 | 0.052 |
| MTS | 0.182 | 1.200 | 1.083–1.329 | <0.001 |
| MTPD | 0.313 | 1.367 | 1.084–1.723 | 0.008 |
| MMS | 0.048 | 1.050 | 0.964–1.143 | 0.263 |
| Osteophyte | 0.489 | 1.631 | 0.864–3.076 | 0.131 |

**Panel B.** Discriminative and classification performance of the standard multivariable logistic regression baseline model

| **Dataset** | **AUC** | **Accuracy** | **Sensitivity** | **Specificity** |
| --- | --- | --- | --- | --- |
| Training set | 0.740 | 0.662 | 0.593 | 0.720 |
| Testing set | 0.706 | 0.654 | 0.563 | 0.737 |

**Abbreviations:** AUC, area under the receiver operating characteristic curve; BMI, body mass index; MTS, medial tibial slope; MTPD, medial tibial plateau depth; MMS, medial meniscal slope; PPV, positive predictive value; NPV, negative predictive value; CI, confidence interval.

**Note:** The standard multivariable logistic regression baseline model was fitted in the training set using the same six predictors as those included in the machine learning analyses: age, BMI, MTS, MTPD, MMS, and posterior tibial osteophytes. Predicted probabilities from the fitted model were then applied to the independent testing set. Classification metrics were calculated using a probability cutoff of 0.5. Posterior tibial osteophyte was coded as present versus absent. Odds ratios and 95% confidence intervals were derived from the training-set logistic regression model.
