# Supplementary figures and images for: Explainable machine learning identifies knee morphology thresholds for arthroscopic medial meniscus posterior root tear: a retrospective cohort study
Source: Front Med (Lausanne). 2026 May 14;13:1819067. doi: 10.3389/fmed.2026.1819067 (PMC13216056; doi:10.3389/fmed.2026.1819067)

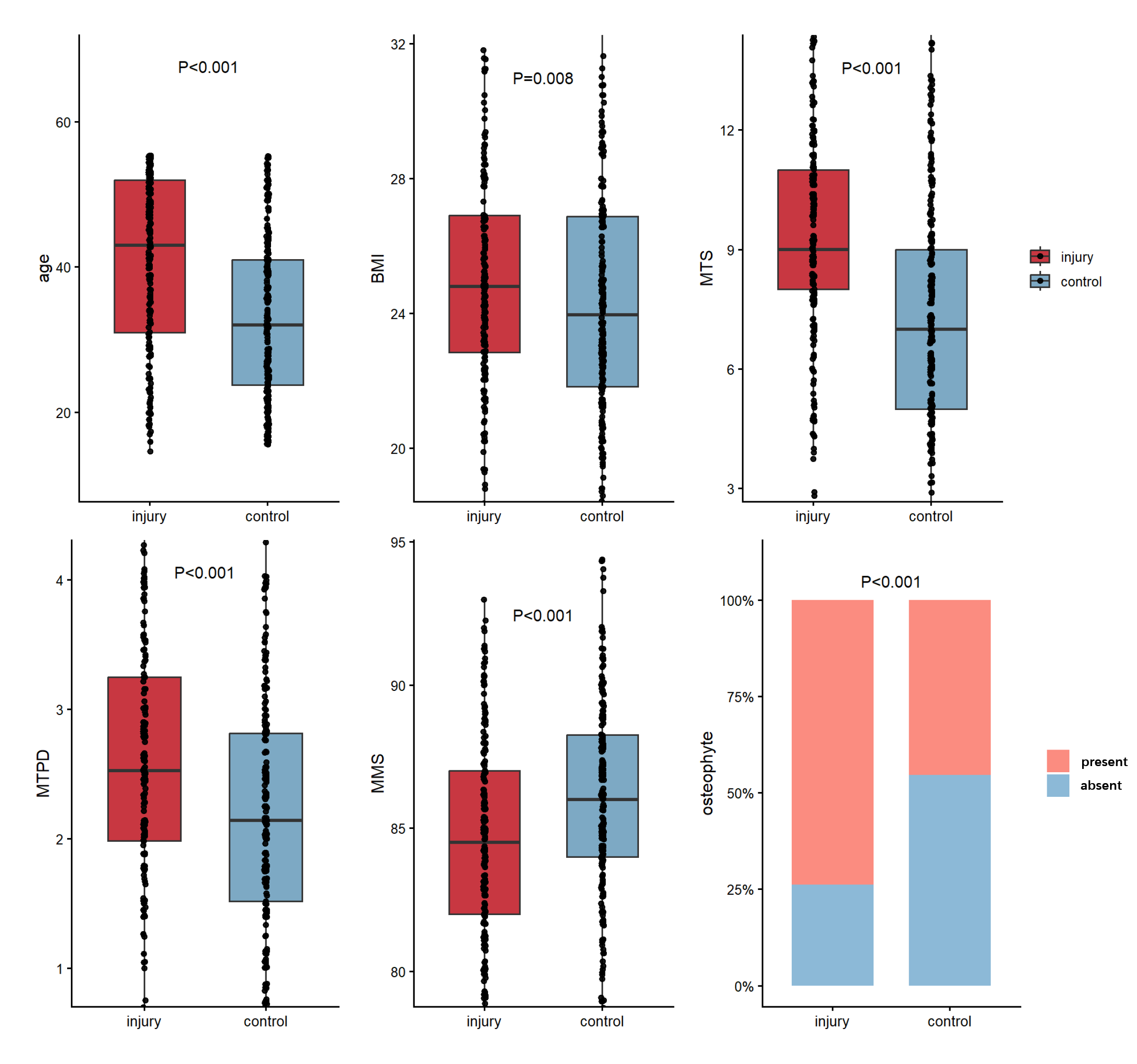

Supplement: Supplementary Figure 1 — Visualization of variables showing statistically significant between-group differences based on the independent-samples t-test and the chi-square test. Box-and-whisker plots were used to display between-group distributions of continuous variables showing statistically significant differences in the training set (age, BMI, MTS, MTPD, and MMS), whereas a stacked bar chart was used to compare posterior tibial osteophytes between groups. BMI, body mass index; MTS, medial tibial slope; MTPD, medial tibial plateau depth; MMS, medial meniscal slope. [file Image_1.TIF]
